# Graphene-supported Pd catalyst for highly selective hydrogenation of resorcinol to 1, 3-cyclohexanedione through giant π-conjugate interactions

**DOI:** 10.1038/srep15664

**Published:** 2015-10-23

**Authors:** Zuojun Wei, Ruofei Pan, Yaxin Hou, Yao Yang, Yingxin Liu

**Affiliations:** 1 Key Laboratory of Biomass Chemical Engineering of the Ministry of Education, College of Chemical and Biological Engineering, Zhejiang University, Hangzhou 310027, China; 2 College of Pharmaceutical Science, Zhejiang University of Technology, Hangzhou 310032, China

## Abstract

The selective hydrogenation of resorcinol (RES) to 1, 3-cyclohexanedione (1,3-CHD) without the addition of alkali is a big challenge. In this article, a novel reduced graphene oxide (rGO) supported Pd catalyst was prepared through co-reduction method, over which we obtained 99.9% of resorcinol conversion and 94.2% of the ever-reported highest 1,3-cyclohexanedione selectivity at 25 °C in only CH_2_Cl_2_ solvent. The excellent selectivity was contributed to the strong π-π and p-π interactions between the graphene nanosheet and the benzene ring as well as hydroxyl in RES molecule. The followed adsorption experiment and Raman analysis also showed the existence of aromatic graphite structures in rGO, which exhibited stronger adsorption towards RES than towards 1,3-CHD.

In recent years, the selective hydrogenation of aromatic compounds has attracted significant research attention because the products obtained from these reactions are usually of high commercial value and hydrogenation is consistently viewed as a clean and facile process. For example, cyclohexanone and cyclohexene derived from hydrogenation of phenol and benzene, respectively, are commercial bulk products used as precursors to produce nylon^1^,^2^,^3^. 1,3-Cyclohexanedione (1,3-CHD), an important chemical intermediate used in the synthesis of pharmaceuticals, pesticides, cosmetics, and polymer additives, can be produced through the selective hydrogenation of resorcinol (RES)^4^,^5^. In theory, the electron density of C = C bonds in the aromatic ring is lower than that in olefins because of the delocalization of strong π bonds in the benzene ring. Thus, electrophilic hydrogenation of aromatic rings is generally more difficult to perform than hydrogenation of olefins (for example, Ea_benzene→cyclohexdiene_ = 70 kJ·mol^−1^; Ea_benzene→cyclohexene_ = 51 kJ·mol^−1^; Ea_cyclohexene→cyclohexane_ = 35 kJ·mol^−1^)^6^,^7^. Therefore, after addition of the first H_2_ molecule to break the aromatic ring, the subsequent addition of the remaining two C = C bonds will be much easier. As a consequence, it is generally difficult to terminate the hydrogenation process at only one or two C = C bonds being added with a high selectivity. For example, the hydrogenation of benzene gives almost zero yield of cyclohexadiene, and the selectivity towards cyclohexene can hardly exceed 90% even with a benzene conversion of only 50%^8^. In past decades, kinds of metal catalysts such as Ru, Pd, or Pt loaded onto different supports, such as active carbon, hydrophilic active carbon, Zeolites, Al_2_O_3_, MgO, and so on, have been tested for the selective hydrogenation of phenol to cyclohexanone^9^,^10^,^11^. However, attaining a high selectivity to cyclohexanone (95%) at elevated conversion of phenol (85%) remains a challenging till in 2009, when Han and his co-workers^12^ achieved excellent conversion (99.9%) and selectivity (99.9%) by using an AlCl_3_-Pd/C dual catalyst. Compared with the break of the benzene ring in phenol by hydrogenation^13^, the benzene ring in RES is more reactive as it is activated by one more adjacent hydroxyl groups through electron-donating effect, which would make the energy barrier of the addition of the first H_2_ molecule be much closer to that of the addition of the remaining two H_2_ molecules. However, it is much more difficult to obtain 1,3-CHD, the one-molecule H_2_ addition product from RES. For example, Wang *et al.*^14^ reported that C_3_N_4_-supported Pd catalyst showed better adsorption of phenols, over which 99% of selectivity toward cyclohexanone were obtained during phenol hydrogenation. While using the same catalyst for RES hydrogenation, however, 1,3-CHD was hard to be produced. Actually, to achieve a high yield of 1,3-CHD in an industrial process, the addition of an alkali or organic amine is always essential and the reaction may follow another pathway^4^.

Although the exact hydrogenation mechanism remains incompletely understood, modern research indicates that the surface properties and the spatial structure of the support perform significant functions in enhancing the selectivity of a reaction^15^,^16^. During hydrogenation of the aromatic ring over a heterogeneous catalyst, it is believed that H_2_ molecules are decomposed into atoms over the metal active cites, while aromatic substrates are adsorbed onto the supports through hydrogen bonds, electrostatic effects and other interaction forces^15^,^16^,^17^. By this means, the aromatic ring is exposed to and attacked by the adsorbed hydrogen atoms and the hydrogenation reaction occurs.

The applications of graphene in materials science, condensed-matter physics^18^, and catalysis science^19^ have been rapidly developed since its first discovery by Geim *et al.* in 2004^20^. Graphene is considered as a new-generation carbon support material because of its excellent physical and chemical properties^21^. Pristine graphene nanosheets generally present large surface areas, hydrophobicity, easy chemical functionalization, and stable structure^22^,^23^. Numerous theoretical studies have identified the presence of π–π interactions between graphene and aromatic rings^24^,^25^,^26^,^27^. For example, Wang’s NMR and adsorption experiments^28^ showed that phenol adsorption increases sharply after the reduction of graphite oxide. Molecular simulations performed by Björk’s group^29^ also confirmed the existence of π–π stacking interactions between graphene nanosheet and aromatic compounds. Inspired by these findings, herein we report the application of graphene supported Pd as a catalyst for the hydrogenation of RES. Since there is strong π–π conjunctive interaction force between graphene nanosheet and RES, and such interaction force will disappear when RES loses its aromatic character after the addition of the first H_2_, the intermediate product with only one molecule H_2_ being added, i.e. the enol isomer of 1,3-CHD, is desorbed from graphene surface and in the end a high selectivity to 1,3-CHD is achieved. The graphene supported Pd catalyst can be reused several times after simple washing with conventional solvents.

## Results and Discussion

We used the Hummers method^30^,^31^,^32^,^33^ to produce graphene oxide and then reduced it together with PdCl_2_ by the addition of NaBH_4_ to obtain stable Pd/graphene (rGO) catalyst containing 3 wt% Pd loading. Catalytic hydrogenation of RES was conducted in a nonaqueous solution, i.e. CH_2_Cl_2_, as shown in Table 1. It can be seen that Pd/rGO catalyst exhibited much higher selectivity to 1, 3-CHD than the other Pd catalysts used in this work: in the experimental ranges, most of the selectivity to 1,3-CHD was more than 80% with a high conversion of RES over Pd/rGO catalyst. Typically, when the reaction took place at 25 °C and 1 MPa H_2_, 1,3-CHD selectivity was as high as 94.2 % with 99.9% conversion of RES after 3 h in the presence of Pd/rGO (entry 3), which is the highest selectivity that has been ever reported. In comparison of Pd/AC catalysis, the selectivity to 1,3-CHD was no more than 30% (entry 8) at the same reaction conditions. Pd/SiO_2_ and Pd/MWCNT catalysts also showed very low selectivity to 1,3-CHD (entries 9 and 10). Since the carbonyl group in 1,3-CHD is not stable under hydrogen environment, high temperature, high pressure or long reaction time could lead to decrease of the selectivity. For instance, 1,3-CHD selectivity was as high as 93.2% over Pd/rGO at 25 °C (entry 2), but dropped to 83.5% at 40 °C (entry 5). 1,3-CHD selectivity decreased from 86.2% (entry 5) to 54.5% (entry 6) at 40 °C as the hydrogenation pressure varied from 0.1 MPa to 2 MPa. With the reaction time varied from 1 h to 3 h, the selectivity to 1,3-CHD also decreased at different temperatures and pressures.

**Figure 1:**
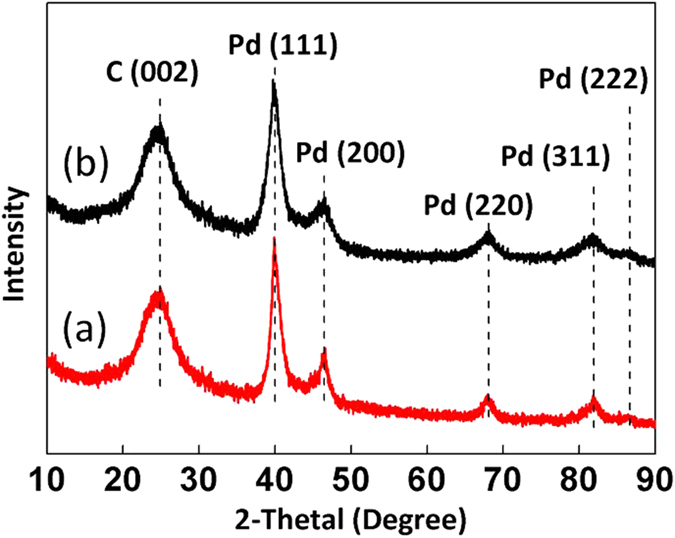
XRD patterns of **(a)** fresh Pd/rGO and **(b)** Pd/rGO after 5 reuses. The diffraction peaks at 40.1°, 46.6°, 68.0°, 82.1° and 86.4° correspond to Pd (111), Pd (200), Pd (220), Pd (311) and Pd (222), respectively. The diffraction peak at 25.1° is ascribed to the 0.335 nm interlayer distance of graphite sheet in the rGO.

**Figure 2:**
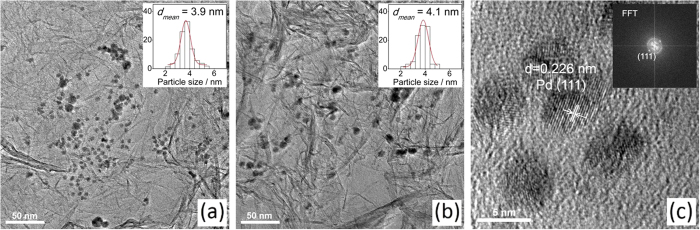
(**a**) TEM image of fresh Pd/rGO, with an average diameter of Pd particles 3.6 nm; **(b,c)** TEM images after 5 reuses, with slight change in the morphology of Pd/rGO and an average diameter of Pd particles 4.1 nm. The insets in **(a,b)** are the particle size distributions of Pd/rGO. The inset in **(c)** is the local fast Fourier transform.

The Pd/rGO catalyst can be reused for at least five times with stable catalytic performance after filtration and washing with CH_2_Cl_2_, at which the selectivity to 1,3-CHD still remained 93.8% with 99.9% conversion of RES (entry 7). Comparison of the XRD (Fig. 1) patterns and TEM (Fig. 2) images of the Pd/rGO catalyst before and after five reaction cycles shows no marked difference in morphology, Pd particle size or distribution. Although some reports argued that unsaturated bonds in graphene can probably be reduced in a hydrogen atmosphere^34^, our Raman spectra (Figure s1) reveal that the graphene structure in the catalysts is not damaged because the reaction conditions used in this work are relatively mild.

**Figure 3:**
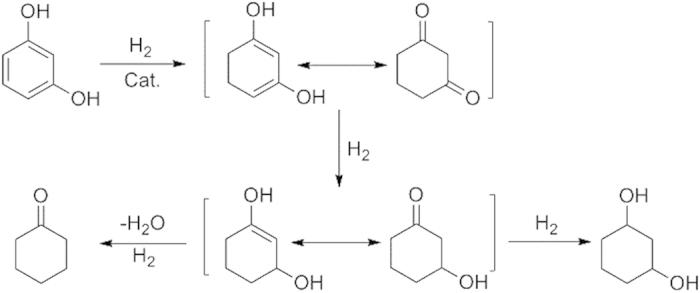
Speculated pathways of the hydrogenation of resorcinol over Pd/rGO catalyst in CH_2_Cl_2_.

GC-MS (Figure s2) detected the main by-products as 3-hydroxy-cyclohexanone, 1,3-cyclohexanediol, and cyclohexanone. Cyclohexanone is mainly produced from the dehydration of 3-hydroxy-cyclohexanone and further addition of 1 mol of hydrogen. The speculated reaction path of RES hydrogenation is shown in Fig. 3.

It is very interesting to note that in Wang’s research^14^, hydrogenation of RES over Pd/C_3_N_4_ produced 99% of cyclohexanone other than 1,3-CHD, while Makowski *et al.*^9^ reported 70% yield of m-hydroxy-cyclohexanone and 30% yield of cyclohexanediol by using another hydrophilic carbon-loading Pd as a catalyst. Two obvious differences may be observed between their work and ours. The first difference involves the solvent used. They preferred to utilize water as a solvent whereas we selected only CH_2_Cl_2_. If other solvents were applied in our reaction system, such as the polar protic water or methanol, or even the polar aprotic acetonitrile or DMSO, the reaction may either not occur or occur with very low activity and selectivity (Table s1). The phenomenon is quite identical to those of Han’s work, in which a success catalytic process was developed for the selective hydrogenation of phenol to cyclohexanone in only nonpolar solvents like CH_2_Cl_2_ and supercritical CO_2_^12^. The second difference involves the polarity of the support. In Wang’s^28^ and Makowski’s work^9^, numerous N atoms within the C_3_N_4_ support and O atoms within hydrophilic carbon may be observed, and these atoms may form hydrogen bonds with hydroxyl and carbonyl groups and interfere with the hydrogenation reaction. In our work, rGO originating from the reduction of graphene oxide by NaBH_4_ was used as a support. The molecular formula of this support is C_6_H_0.75_O_0.14_ (entry 1, in Table s2) with the oxygen content of around 3 w%, indicating that most of the polar functional groups have been removed such that the resultant graphene nanosheets are completely hydrophobic. Different hydrogenation products were obtained due to the discrepancies of the solvent and support, indicating that different catalytic mechanism was followed^35^,^36^,^37^,^38^,^39^,^40^. Wang^14^ attributed the excellent selectivity of the Pd/C_3_N_4_ catalyst to phenol forming strong interaction force with nitrogen in the support through hydrogen-bond interactions while cyclohexanone cannot. As a consequence, the intermediate product cyclohexanone is more likely to desorb out from the support and thus the excessive hydrogenation is prevented. In our case, the hydrophobic support of rGO can hardly form hydrogen bonds. We therefore suppose that the excellent selectivity observed is due to the π–π and p–π conjugation interactions of giant π bond on the graphene nanosheets with the aromatic ring and hydroxyl in RES. According to this speculation, we can easily determine that the polar solvents we have tested are not appropriate for the selective hydrogenation of RES because of their negative impacts on π–π and p–π interactions. To further verify this thought, we prepared rGO-E and rGO-H by reducing graphene oxide using ethylene glycol^41^,^42^ and hydrazine hydrate^43^, respectively, as reducing agents. Both the supports supported Pd catalysts exhibited poor performance for RES hydrogenation because rGO-E (with formula of C_6_H_2.45_O_1.39_, in Table s2) contains more than 23 w% of hydrophilic oxygen atoms as ethylene glycol cannot reduce graphene oxide completely and hydrazine hydrate introduces 4 w% of hydrophilic nitrogen atoms into the support (Table 2s), which will also interfere in the π–π and p–π interactions between graphene surface and the substrate.

Further proof comes from the adsorption tests of RES and 1,3-CHD on different supports. As shown in Figure s3 and s4, both the adsorption of RES and 1,3-CHD on rGO reached equilibrium after 20 min, and the equilibrium adsorption capacities toward RES and 1,3-CHD were 0.072 and 0.039 g·g^−1^, respectively. The corresponding selectivity coefficient was 1.89–2.02 (Table 2), which means that RES adsorption occurs more extensively than 1,3-CHD adsorption. Although SiO_2_ showed large surface area, and its adsorption capacities to RES and 1,3-CHD were larger than those of rGO, the corresponding selectivity coefficient approached 1.00, indicating no adsorption selectivity between RES and 1,3-CHD was observed. AC also showed large adsorption capacities to RES and 1,3-CHD, but the selectivity coefficient was only 1.16–1.21. In contrast, MWCNT showed favorable adsorption selectivity toward RES (selectivity coefficient 1.58–1.65, in Table 2) which is comparable to that of rGO, indicating that the giant π bonds of graphite structure in these two supports have strong interaction forces with RES.

**Figure 4:**
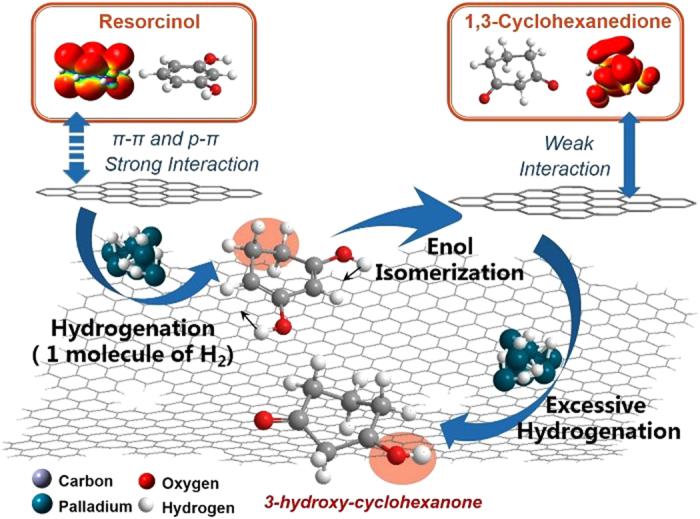
Plausible catalytic mechanism for the hydrogenation of resorcinol to 1,3-cyclohexanedione over Pd/rGO.

It is well known that a heterogeneous catalytic reaction starts with the adsorption of the substrate onto the support and followed by reaction at the catalytic active center. Our adsorption experiment shows that graphite structures exhibit stronger adsorption toward RES due to the π–π and p–π conjugate interactions between them, which means that RES is superior to 1,3-CHD in occupying active sites. After addition of 1 mol of hydrogen to RES, 1,3-CHD (enol isomer) was generated, and its less interaction force with graphene surface allows it to leave away easily. Thus, excessive hydrogenation is prevented and the selectivity toward 1,3-CHD is enhanced. As a result, Pd/rGO showed the highest selectivity to 1,3-CHD (94.2%, entry 3) and Pd/SiO_2_ showed the lowest selectivity to 1,3-CHD (21.1%, entry 9) since SiO_2_ shows no difference in the adsorption of RES and 1,3-CHD. The catalytic mechanism is illustrated as in Fig. 4.

**Figure 5:**
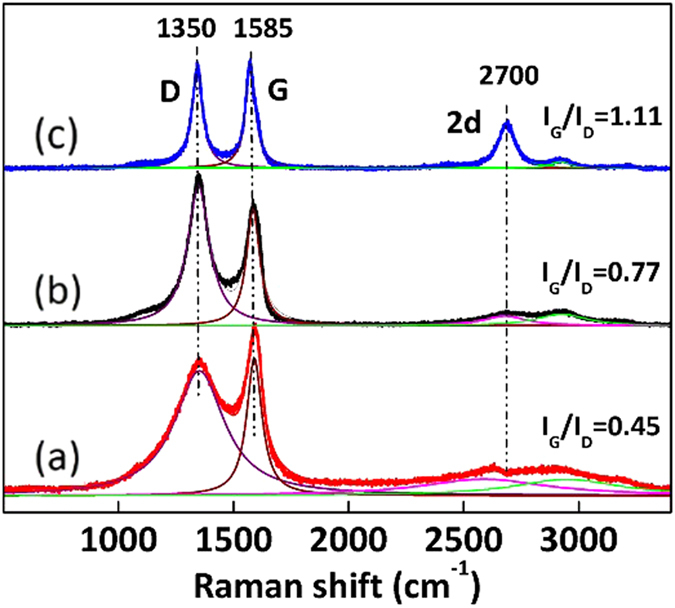
Raman spectra of various carbon materials. **(a)** Activated carbon; **(b)** rGO; **(c)** Multi-walled carbon nanotube.

The graphitization degree of a carbon material is generally characterized by the I_G_/I_D_ value in the Raman spectra in which I_G_ is the intensity of G band around 1575 cm^−1^ corresponding to sp^2^-bonded carbon atoms in a hexagonal graphitic ring, and I_D_ is the intensity of D band around 1350 cm^−1^ corresponding to the vibrations of sp^3^-bonded carbon atoms of defects and disorder. As shown in Fig. 5, the I_G_/I_D_ order of the carbon materials is: MWCNT > rGO > AC, indicating that MWCNT would possess the highest adsorption selectivity coefficient and be the best support for the selectivity hydrogenation of RES to 1,3-CHD, which is somewhat contradict to our experimental data. As the particle sizes and dispersion of Pd in rGO and MWCNT do not vary so much that may affect the selectivity (Table 2), we deduce the main reason may be that MWCNT containing the bending graphite surface weakens the interaction of its giant π bonds with other aromatic compounds, resulting in a lower adsorption selectivity to RES and lower reaction selectivity to 1,3-CHD than those of rGO^44^,^45^. Other carbon materials with more perfect graphite structure^26^,^46^ are also under developing for the selective hydrogenation of aromatic compounds in our lab.

## Conclusions

In conclusion, Pd/rGO catalyst was prepared by co-reduction of graphene oxide and PdCl_2_ with NaBH_4_. The as-prepared novel catalyst showed high activity and selectivity for the hydrogenation of RES to 1,3-CHD, which could be attributed to the π–π or p–π strong interaction between the giant π bonds on the graphene nanosheets and the aromatic ring and hydroxyls in RES.

## Methods

### Chemicals and Materials

All reagents were obtained from Sino-pharm Chemical Reagent Co. Ltd, except that PdCl_2_ was purchased from Shanghai Jiuling Chemical Co. Ltd.

### Synthesis of graphene oxide (GO)

Graphene oxide was synthesized from natural graphite powder by using a modified Hummers method consisting of two steps of oxidation. In the first pre-oxidation step, concentrated H_2_SO_4_ (40 mL), K_2_S_2_O_7_ (8.4 g), and P_2_O_5_ (8.4 g) were added into a 500 mL round-bottom flask and maintained at 80 °C for 4.5 h. Then the mixture was cooled to room temperature, diluted with deionized water, left overnight, vacuum-filtered, and washed with deionized water (1.6 L) to obtain the pre oxidized material. In the second oxidation step, concentrated H_2_SO_4_ (230 mL) and the pre oxidized material were added into a 1 L three-necked round-bottom flask and chilled to 0 °C. KMnO_4_ (60 g) was added carefully under continuous stirring to keep the reaction temperature below 10 °C for 30 min. Afterwards, the reaction temperature was gradually increased to less than 35 °C and maintained for 2 h. The mixture was diluted with deionized water (0.5 L) and stirred for 2 h, successively diluted with an additional deionized water (1.5 L), drop wisely added with H_2_O_2_ (25 mL), and left undisturbed for 4 days. The precipitate was washed with HCl (1 mol·L^−1^) and centrifuged for at least three recycles to remove residual metal oxides, and repeatedly washed with deionized water and centrifuged until the filtrate became neutral. Finally, the brown mixture dispersion in water was sonicated for 30 min, centrifuged, and freeze-dried to obtain the final GO.

### Synthesis of Pd/rGO

1 g of GO was dispersed through sonication in 200 ml of deionized water. 52 mg of PdCl_2_ was added and the mixture was rigorously stirred for 1 h. After that, 100 mL of NaBH_4_ aqueous solution (7.5 g·L^−1^) was drop wisely added in and the mixture was incubated at 80 °C for 4 h. The resulting Pd/rGO catalyst was then filtrated and vacuum dried for use. The Pd content in the catalyst was determined to be 3.1 wt% by Atomic Absorption Spectrometer (AAS, Agilent AA240).

### Synthesis of Pd/rGO-E

1 g of GO was dispersed through sonication in 200 ml of ethylene glycol-water solution (3:2, v/v). 52 mg of PdCl_2_ was added and the mixture was rigorously stirred for 1 h. After that, the mixture was incubated at 120 °C for 4 h. The resulting Pd/rGO-E catalyst was then filtrated and vacuum dried for use. The Pd content in the catalyst was determined to be 3.0 wt% by AAS.

### Synthesis of Pd/rGO-H

1 g of GO was dispersed through sonication in 200 mL of 1 w% N_2_H_4_ aqueous solution. 52 mg of PdCl_2_ was added and the mixture was rigorously stirred for 1 h. After that, the mixture was incubated at 95 °C for 8 h. The resulting Pd/rGO-H catalyst was then filtrated and vacuum dried for use. The Pd content in the catalyst was determined to be 3.1 wt% by AAS.

### Synthesis of Pd/X (X = activated carbon (AC), multi-walled carbon nanotube (MWCNT), and SiO_2_)

Pd/X was prepared by wetness impregnation method. Taking Pd/AC as an example, 1 g of AC was wetness impregnated by PdCl_2_ acidic aqueous solution containing 5 mg·mL^−1^ of Pd (II). The impregnation process was repeated for several times till the calculated Pd content in the final catalyst reached ca 3 wt%. The solid was finally vacuum dried at 120 °C and reduced by hydrogen at 350 °C for 4 h to obtain the final Pd/AC catalyst. The Pd contents in Pd/AC, Pd/MWCNT and Pd/SiO_2_ catalysts were 3.1 wt%, 3.0 wt% and 2.9 wt%, respectively, determined by AAS.

### Characterization

The X-ray powder diffraction (XRD) patterns of the catalysts were measured with a Thermo X’TRA X-ray diffractometer using CuKα radiation (40 kV, 40 mA). Transmission electron microscopy (TEM) images were obtained using a Tecnai G2 F30 S-Twin instrument (FEI Co.) operating at an accelerating voltage of 300 kV. Raman spectra were collected on a Jobin-Yvon LabRam HR 800 Raman spectroscope equipped with a 514.5 nm laser source. BET specific surface areas and pore structures were measured by pulsed nitrogen adsorption-desorption method at −196 °C using an ASAP 2010 instrument (Micromeritics Instrument Co.). Pd content in a catalyst was determined by Atom Absorption Spectroscopy AA240 (Agilent Technologies). The elemental composition of rGO was determined by Leco-Chns932 (LECO Co.). The dispersion of Pd was obtained by CO chemisorption method, which was carried out at 40 °C on a Quantachrome Autosorb-1/C chemisorb apparatus. Prior to measurements, the pre-reduced catalysts were reduced *in situ* for 2 h at 450 °C in H_2_. The metal dispersions were estimated based on an adsorption stoichiometry Pd/CO = 1.5 according to Han *et al.*’s work^47^, which were close to those obtained from TEM^36^.

### Typical procedure for hydrogenation of resorcinol

The reaction was carried out in a stainless batch reactor (20 mL) with a magnetic stirrer. In a typical procedure, resorcinol (0.027 mmol), catalyst (50 mg, ca 3 wt% of Pd), and 5 ml of CH_2_Cl_2_ was added in the reactor. After purging 3 times with H_2_, the reactor was heated and pressurized with H_2_ to the desire temperature and pressure to initiate the reaction. After reaction the reactor was placed in ice water to quench the reaction and the products were analyzed by GC-MS.

### Typical procedure for adsorption of resorcinol and 1,3-cyclohexanedione

Taking the adsorption of rGO as an example. 12 mL, 8 mL and 4 mL of CH_2_Cl_2_ containing 3 mg·mL^−1^ of resorcinol (RES) and 1,3-cyclohexanedione (1,3-CHD) were introduced to three quartz tubes (25 mL), respectively, followed by adding 50 mg of rGO into each of the tubes. The tubes were then incubated at 25 °C under sonication. Samples were taken at intervals till the adsorption reached equilibrium.

The adsorption selectivity factor of resorcinol to 1,3-cyclohexanediene was estimated as:


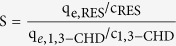


Where q_e_ is the equilibrium adsorption amount of the solute in the adsorbent, and c is the equilibrium concentration of solute in CH_2_Cl_2_ solvent.

### GC-MS analysis

Reaction products were qualitatively measured by an Agilent 6890–5973 GC-MS instrument. The chromatographic separation was performed using a HP-5 capillary column (30.0 m × 0.25 mm × 0.32 μm). Helium (99.999%) was used as gas carrier with a constant flow rate of 2.0 mL·min^−1^ and a 1:10 split ratio. The oven was heated using the following temperature curve: initial temperature of 100 °C, heated up to 280 °C at the rate of 12 °C·min^−1^ and kept for 10 min. The vaporizing chamber temperature was 280 °C and the temperature of the GC/MS interface was kept at 300 °C. The mass spectrometer was operated in EI mode at 70 V of ionization voltage, 0.8 kV of detector, temperatures of ion source of 280 °C and quadrupole of 150 °C.

To quantitatively measure the reaction components, an Agilent 6820 GC equipped with HP-5 capillary column (30.0 m × 0.25 mm × 0.32 μm) and a flame ionization detector was used. n-Dodecane was used as an internal standard.

## Additional Information

**How to cite this article**: Wei, Z. *et al.* Graphene-supported Pd catalyst for highly selective hydrogenation of resorcinol to 1,3-cyclohexanedione through giant π-conjugate interactions. *Sci. Rep.* **5**, 15664; doi: 10.1038/srep15664 (2015).

## Supplementary Material

Supplementary Information

## Figures and Tables

**Table 1:** Hydrogenation of resorcinol over Pd catalysts in CH_2_Cl_2_ solvent.

Entry	Catalysts	T (°C)	P_H2_(MPa)	Time (h)	RES Con. (%)	1,3-CHD Sel. (%)
1	Pd/rGO	20	0.1	3	84.0	100
2	Pd/rGO	25	0.5	3	99.9	93.2
3	Pd/rGO	25	1	1	95.1	99.9
				3	99.9	94.2
4	Pd/rGO	30	0.5	1	83	90.7
				2	92.1	87.1
				3	99.9	85.1
5	Pd/rGO	40	0.5	0.5	70.6	86.2
				1	99.9	83.5
6	Pd/rGO	40	2	0.5	99.9	54.5
7^a^	Pd/rGO	25	1	3	99.9	93.8
8	Pd/AC	25	1	1	99.9	26.6
9	Pd/SiO_2_	25	1	3	99.9	21.2
10	Pd/MWCNT	25	1	3	99.9	52.3

Reaction conditions: resorcinol (0.027 mmol), CH_2_Cl_2_ solvent (3 ml), 50 mg of Pd catalyst with 3 wt% Pd loading. ^a^The 5th reuse of Pd/rGO.

**Table 2:** Surface character of various catalysts and their adsorption properties towards resorcinol and 1,3-CHD.

Catalysts	Pd particle size (nm)		Metal dispersion (%)^a^	Specific surface area (m^2^·g^−1^)	Saturated adsorption amount(mg·g^−1^)		Selectivity coefficient of RES to 1,3-CHD
	TEM	XRD			RES	1,3-CHD	
Pd/SiO_2_	5.0	5.6	/	157	135 ± 2.7	135 ± 2.8	0.99–1.01
Pd/AC	4.8	6.3	/	1499	205 ± 4.9	193 ± 4.6	1.16–1.21
Pd/MWCNT	4.5	7.2	25.4	351	102 ± 2.2	57 ± 1.3	1.58–1.65
Pd/rGO	4.1	5.9	27.9	77.4	72 ± 1.6	39 ± 0.9	1.98–2.02

**Note:** The saturated adsorption amount is estimated as the maximum adsorption amount of the solute with the increase in its concentration in CH_2_Cl_2_ solvent.

^a^Metal dispersion was estimated through CO-TPD by assuming the adsorption stoichiometry of Pd/CO = 1.5^47^.
