# Supplementary material for: Graphene-supported Pd catalyst for highly selective hydrogenation of resorcinol to 1, 3-cyclohexanedione through giant π-conjugate interactions
Source: Sci Rep. 2015 Oct 23;5:15664. doi: 10.1038/srep15664 (PMC4616164; doi:10.1038/srep15664)
Supplement: Supplementary Information [file srep15664-s1.doc]

**Graphene-supported Pd catalyst for highly selective hydrogenation of Resorcinol to 1,3-cyclohexanedione through giant π-conjugate interactions**

**Supporting Information**

Zuojun Wei,*^,a^ Ruofei Pan,^a^ Yaxin Hou,^a^ Yao Yang,^a^ Yingxin Liu^b^

^a^ Key Laboratory of Biomass Chemical Engineering of the Ministry of Education, College of Chemical and Biological Engineering, Zhejiang University, Hangzhou 310027, China

^b^ College of Pharmaceutical Science, Zhejiang University of Technology, Hangzhou 310032, China.

* Corresponding author, Email: weizuojun@zju.edu.cn

**Contents**

[Table s1 Effect of solvents on the hydrogenation of resorcinol over Pd/rGO catalyst 3](#__RefHeading___Toc430377185)

[Table s2 Effect of the rGO support on the hydrogenation of resorcinol over Pd/rGO catalyst 3](#__RefHeading___Toc430377186)

[Table s3 Surface characteristics of Pd-based catalysts with different supports 4](#__RefHeading___Toc430377187)

[Figure s1 Raman spectra of Pd/rGO (a) before use; (b) after 5-time reuse 5](#__RefHeading___Toc430377188)

[Figure s2 GC-MS spectra of components detected during the hydrogenation of resorcinol 7](#__RefHeading___Toc430377189)

[Figure s3 Kinetic curves of single-component adsorption curve of resorcinol and 1,3-CHD on rGO 8](#__RefHeading___Toc430377190)

[Figure s4 Adsorption curves of resorcinol and 1,3-CHD on (a) rGO, (b) MWCNT, (c) AC, and (d) SiO_2_ 9](#__RefHeading___Toc430377191)

[Figure s5 Nitrogen isotherm adsorption-desorption curves of (a) Pd/rGO, (b) Pd/MWCNT, (c) Pd/AC and (d) Pd/SiO_2_ 10](#__RefHeading___Toc430377192)

[Figure s6. TEM images of (a) Pd/AC; (b) Pd/SiO_2_ and (c) Pd/MWCNT. 11](#__RefHeading___Toc430377193)

[Figure s7. XRD patterns of (a) Pd/SiO_2_; (b) Pd/MWCNT and (c) Pd/AC. 11](#__RefHeading___Toc430377194)

# Table s1 Effect of solvents on the hydrogenation of resorcinol over Pd/rGO catalyst

| Entry | T  ( ^o^C) | | Time  (h) | Solvents | Conversion of RES  (%) | Selectivity to 1,3-CHD (mol%) |
| --- | --- | --- | --- | --- | --- | --- |
| 1 | | 25 | 1 | CH_3_CN | 0 | N.D. |
|  | |  | 3 | CH_3_CN | 0 | N.D. |
| 2 | | 60 | 4 | CH_3_CN | 0 | N.D. |
| 3 | | 60 | 4 | H_2_O | 79 | N.D. |
| 4 | | 25 | 3 | CH_3_OH | 3.6 | N.D. |
|  | |  | 6 | CH_3_OH | 10.6 | N.D. |
| 5 | | 25 | 4 | C_2_H_4_Cl_2_ | 99.9 | 26.3 |
| 6 | | 25 | 6 | C_2_H_4_Cl_2_ | 99.9 | 20.0 |

Reaction conditions: 0.027 mmol of resorcinol, 3 ml of solvent, 50 mg of Pd/rGO catalyst and 1 MPa of H_2_.

# Table s2 Effect of the rGO support on the hydrogenation of resorcinol over Pd/rGO catalyst

| Entry | Support | | | RES Conversion  (%) | 1,3-CHD Selectivity  (%) |
| --- | --- | --- | --- | --- | --- |
|  | Name | Reductant | Formula of rGO |  |  |
| 1 | rGO | NaBH_4_ | C_6_H_0.75_O_0.14_ | 99.9 | 94.2 |
| 2 | rGO-E | HOCH_2_CH_2_OH | C_6_H_2.45_O_1.39_ | 18.9 | 37.3 |
| 3 | rGO-H | N_2_H_4_ | C_6_H_0.79_O_0.2_N_0.23_ | 23.5 | 86.7 |

Reaction conditions: 0.027 mmol of resorcinol, 3 ml of CH_2_Cl_2_, 25 ºC of temperature, 50 mg of Pd catalyst, 1 MPa of H_2_ and 3 h of reaction time.

# Table s3 Surface characteristics of Pd-based catalysts with different supports

| Sample | S_BET_ (m^2^⋅g^-1^) | V (cm^3^⋅g^-1^) | Median pore diameter (nm) |
| --- | --- | --- | --- |
| Pd/rGO | 80 | 0.095 | 4.71 |
| Pd/MWCNT | 111 | 0.518 | 18.73 |
| Pd/AC | 1495 | 0.907 | 2.43 |
| Pd/SiO_2_ | 152 | 0.080 | 2.13 |


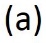

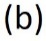

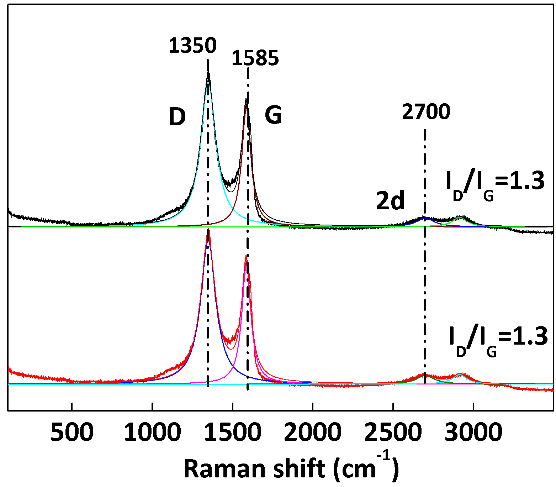


# Figure s1 Raman spectra of Pd/rGO (a) before use; (b) after 5-time reuse


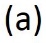

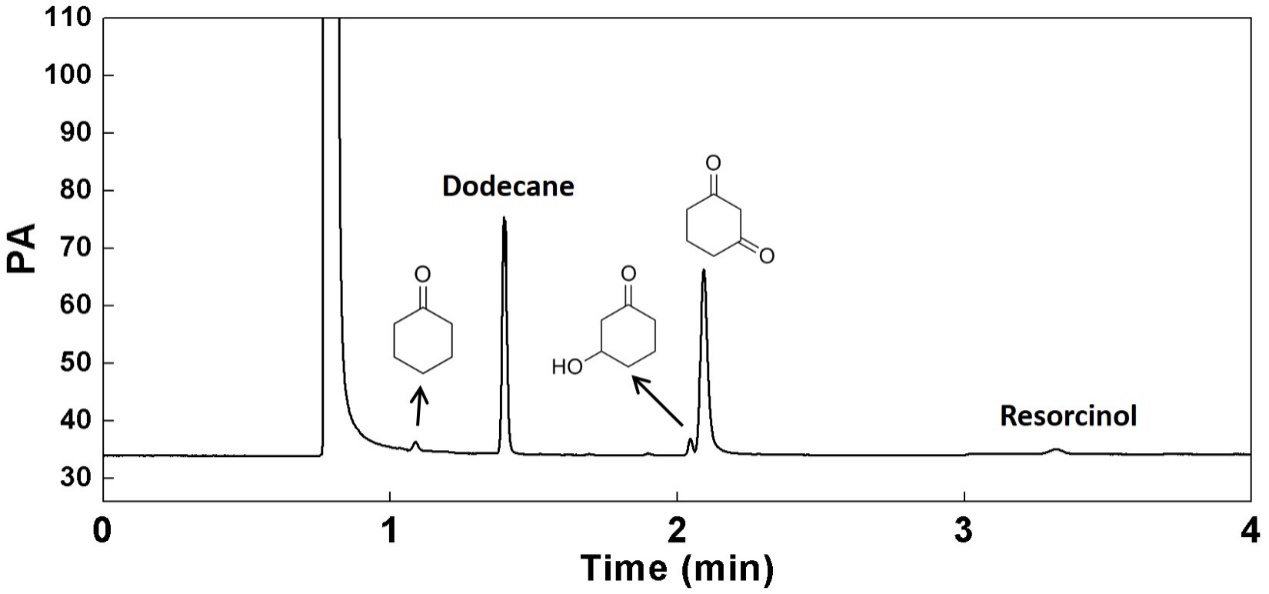


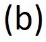

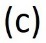

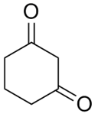

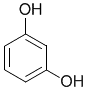

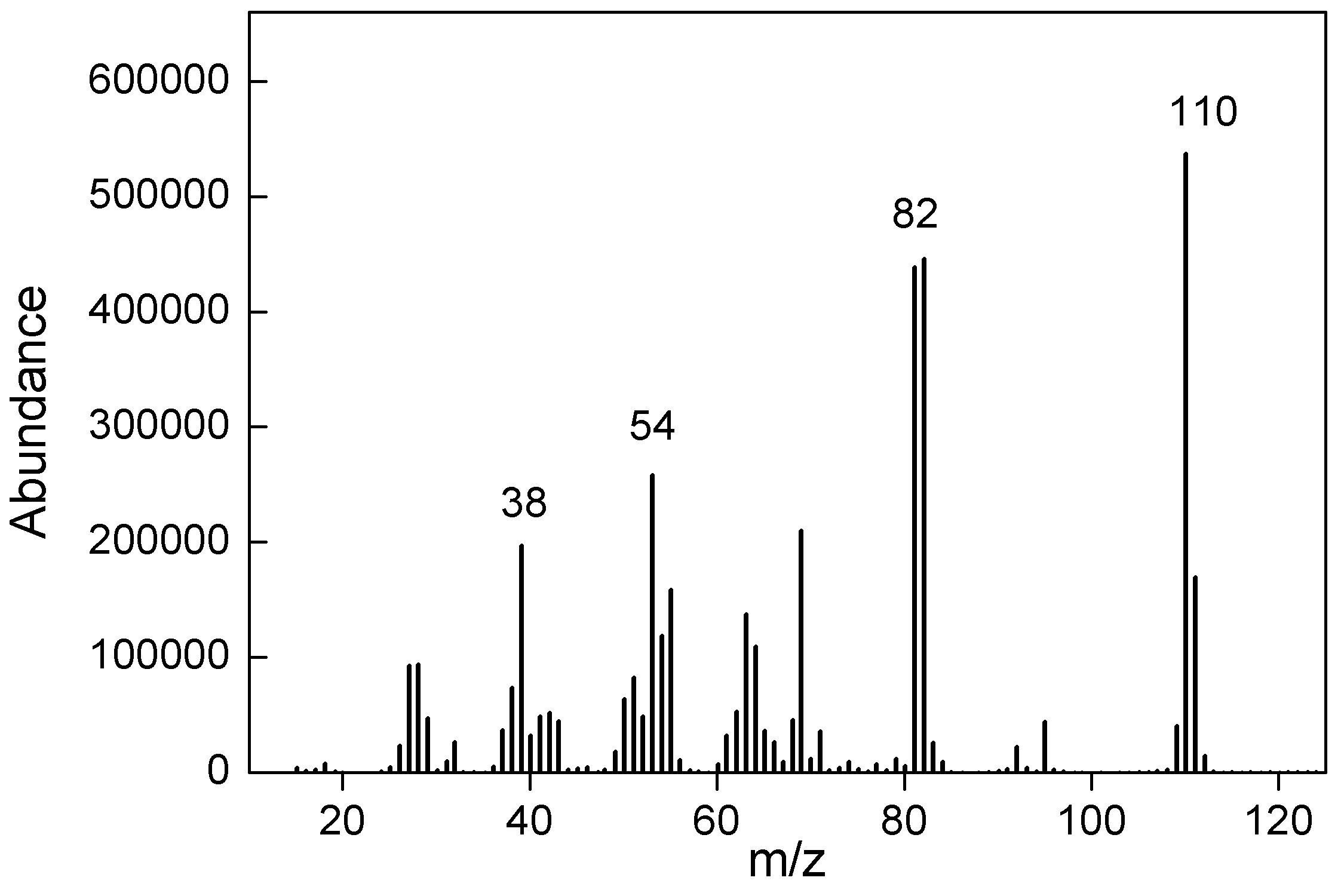

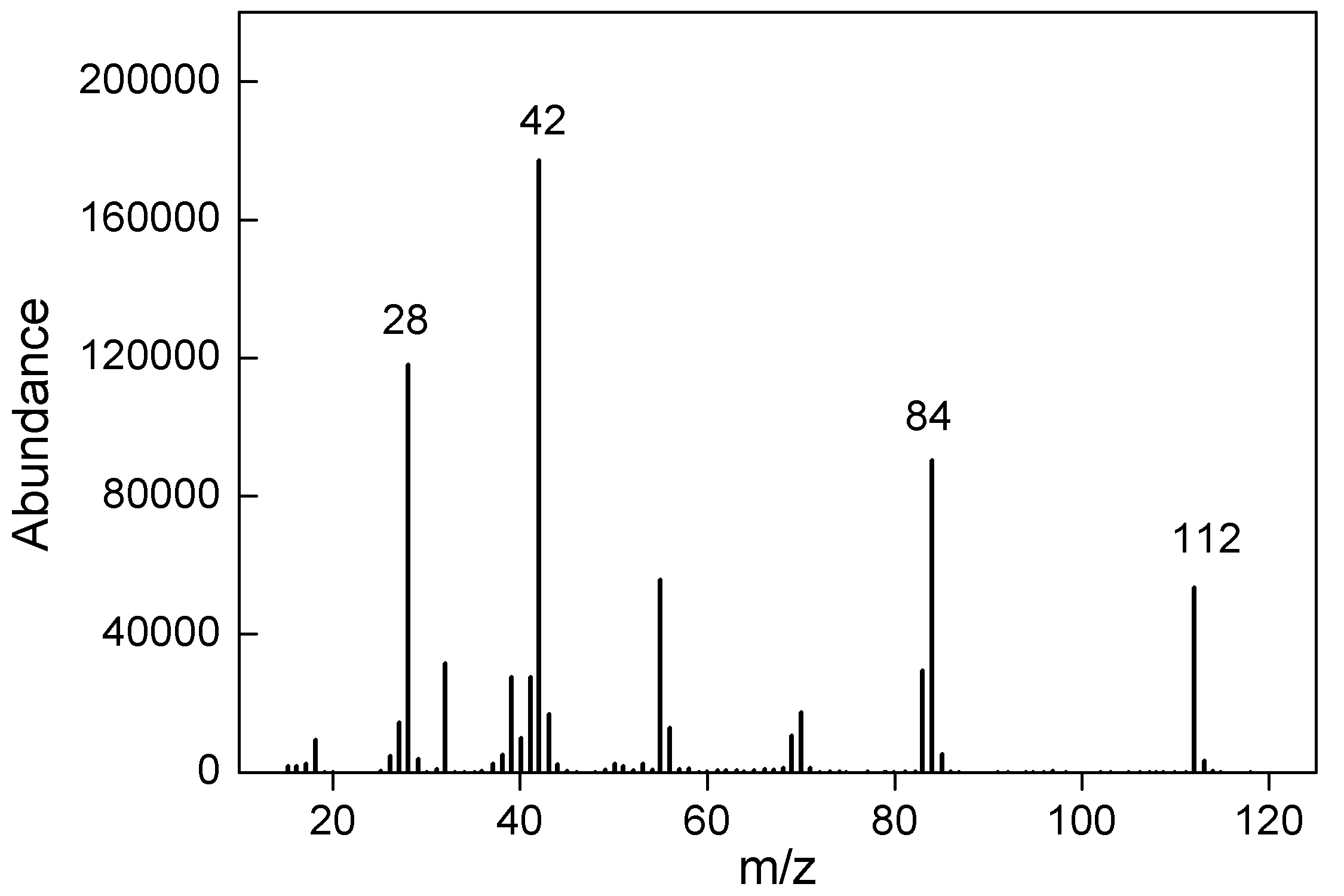


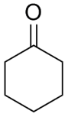

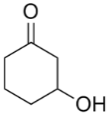

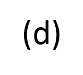

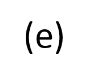

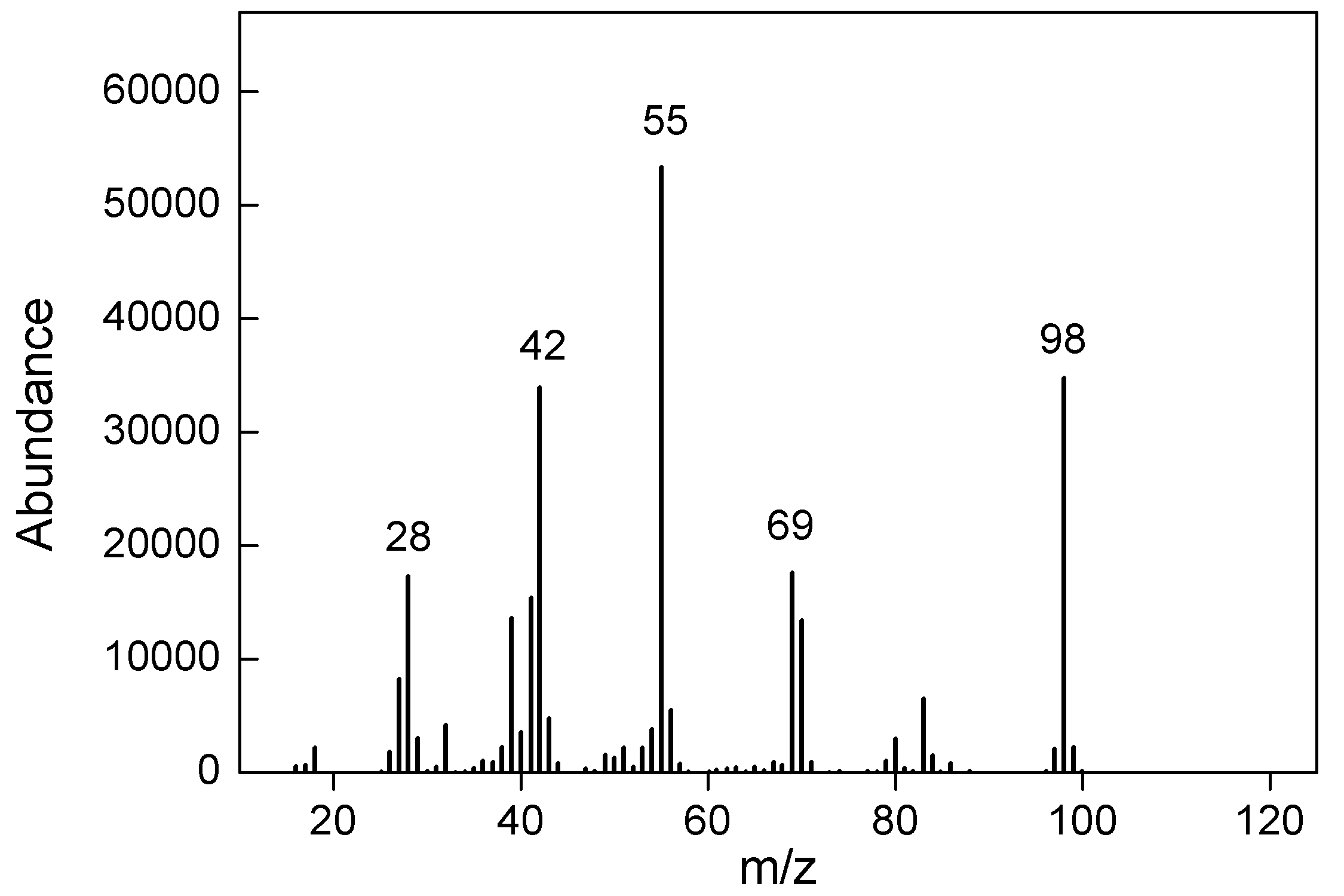

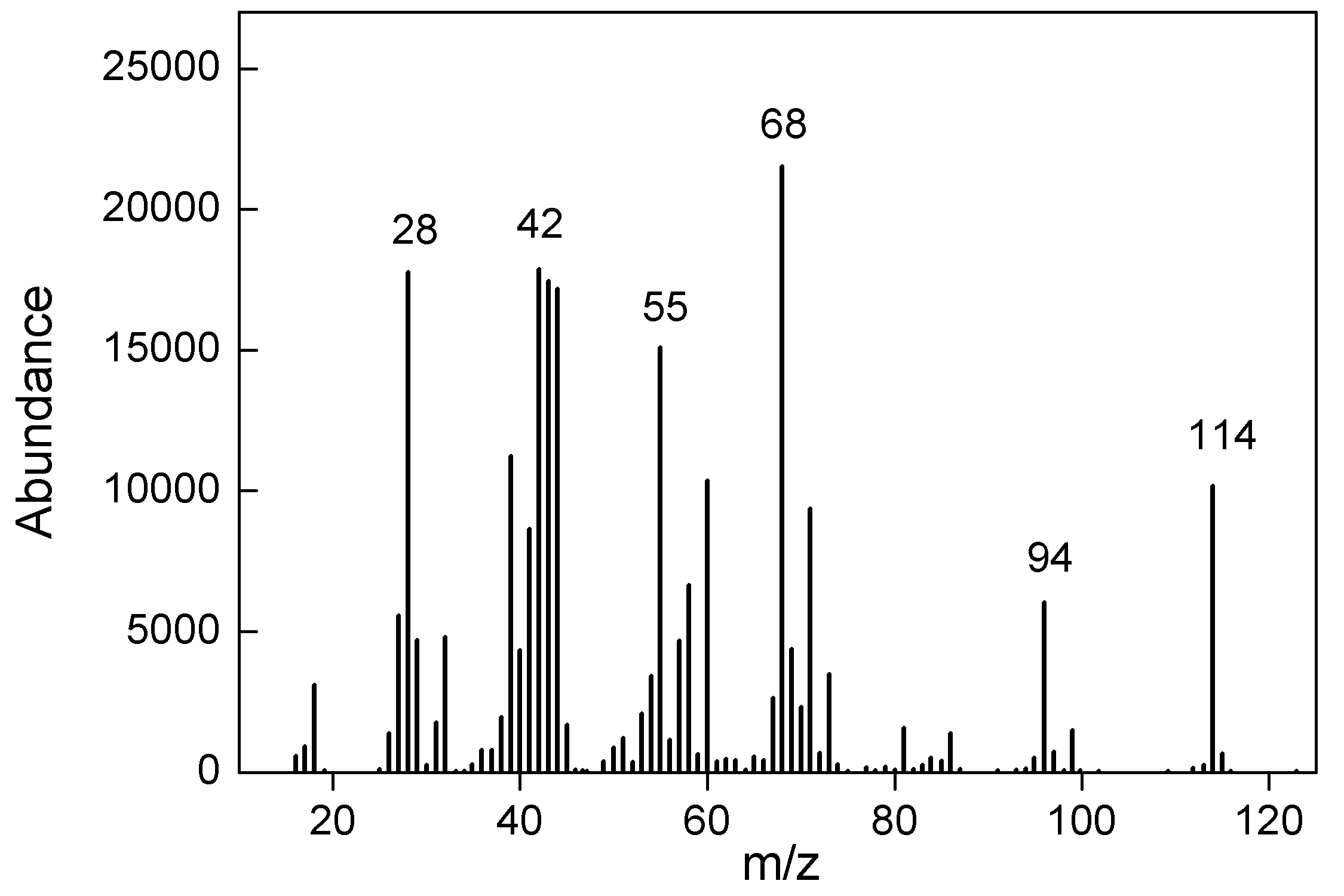


# Figure s2 GC-MS spectra of components detected during the hydrogenation of resorcinol. (a) GC spectra; MS spectra of (b) resorcinol, (c) 1,3-cyclohexanedione, (d) cyclohexanone and (e) 3-hydroxy-cyclohexanone


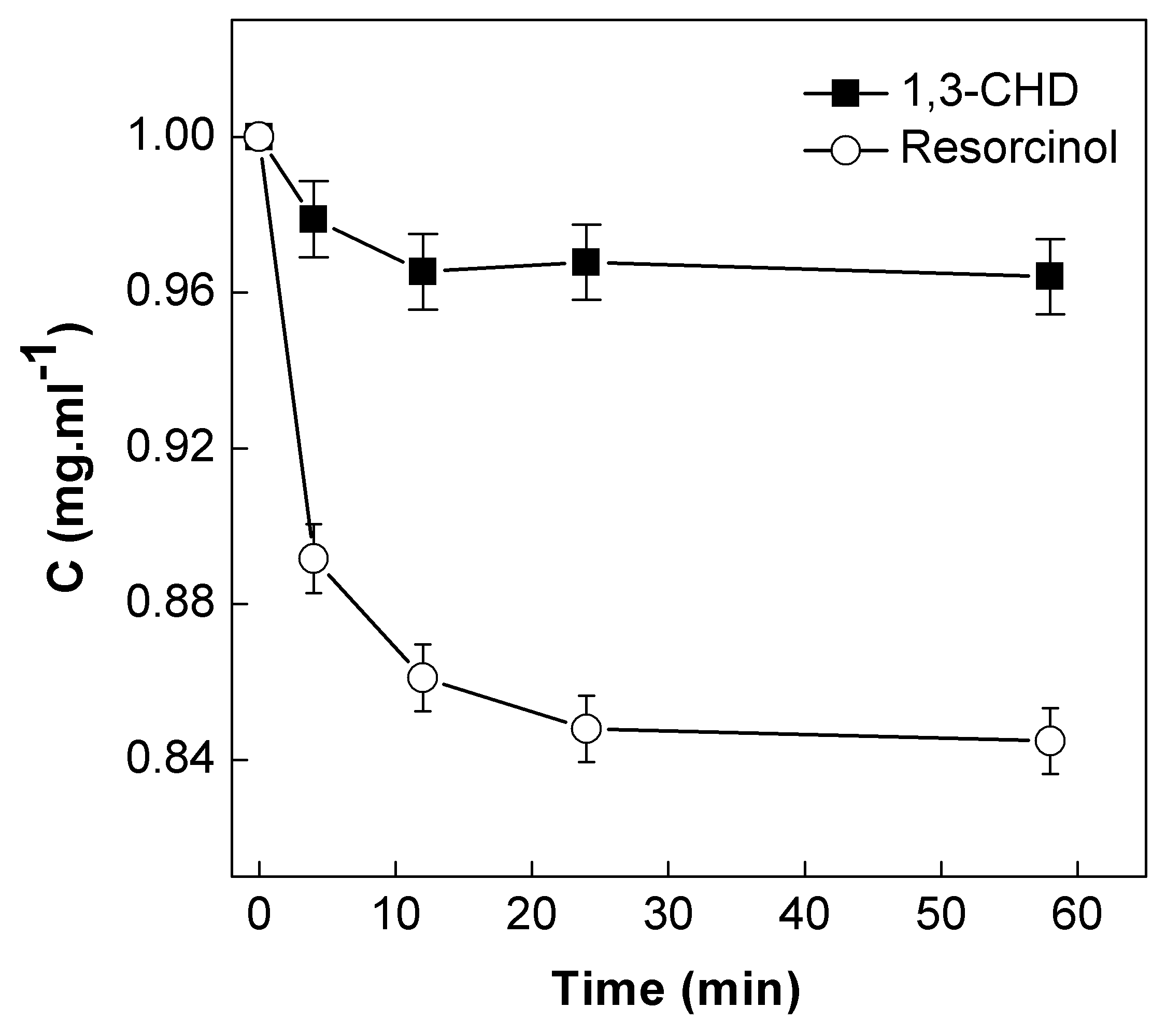


# Figure s3 Kinetic curves of single-component adsorption curve of resorcinol and 1,3-CHD on rGO


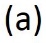

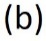

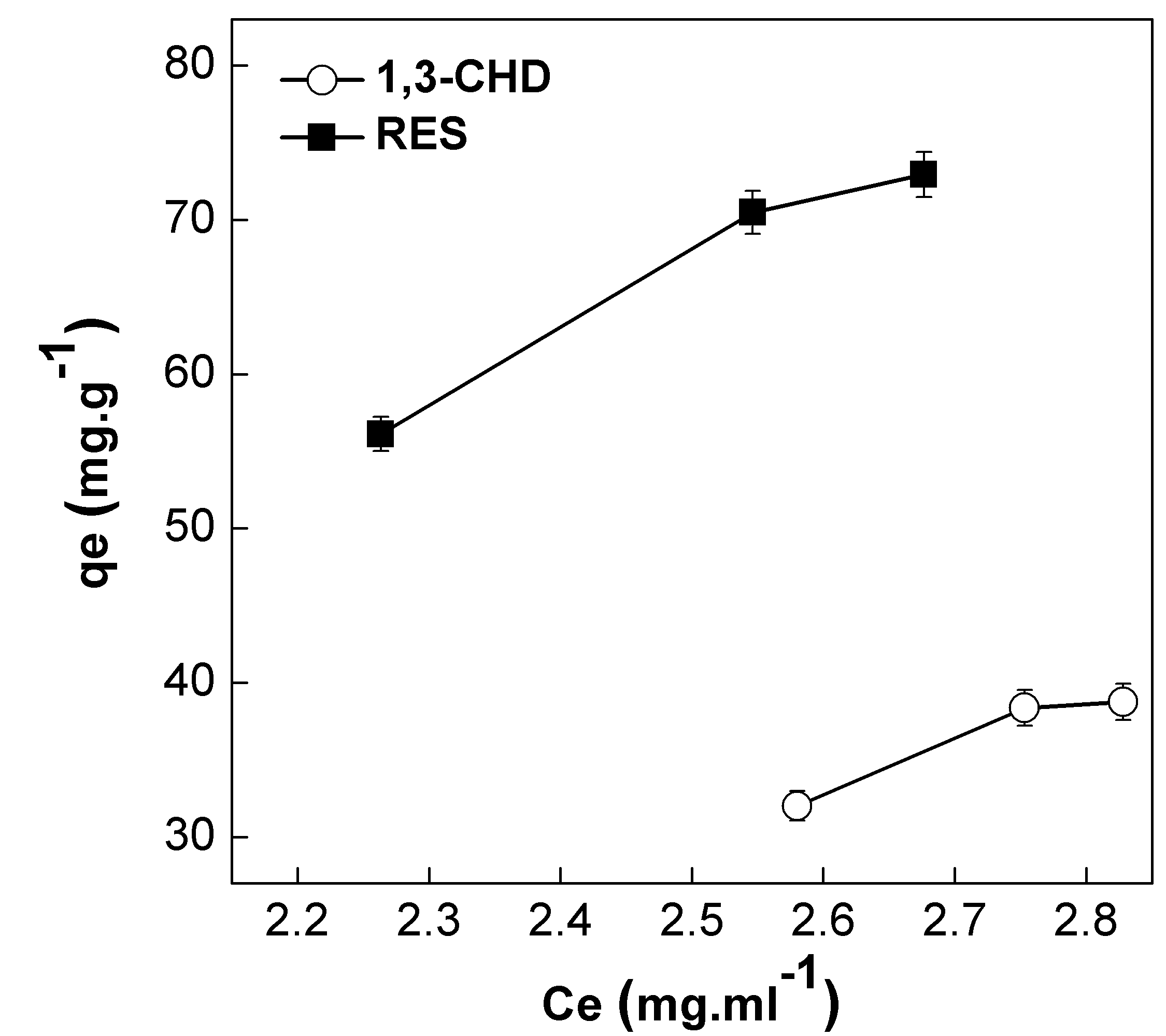

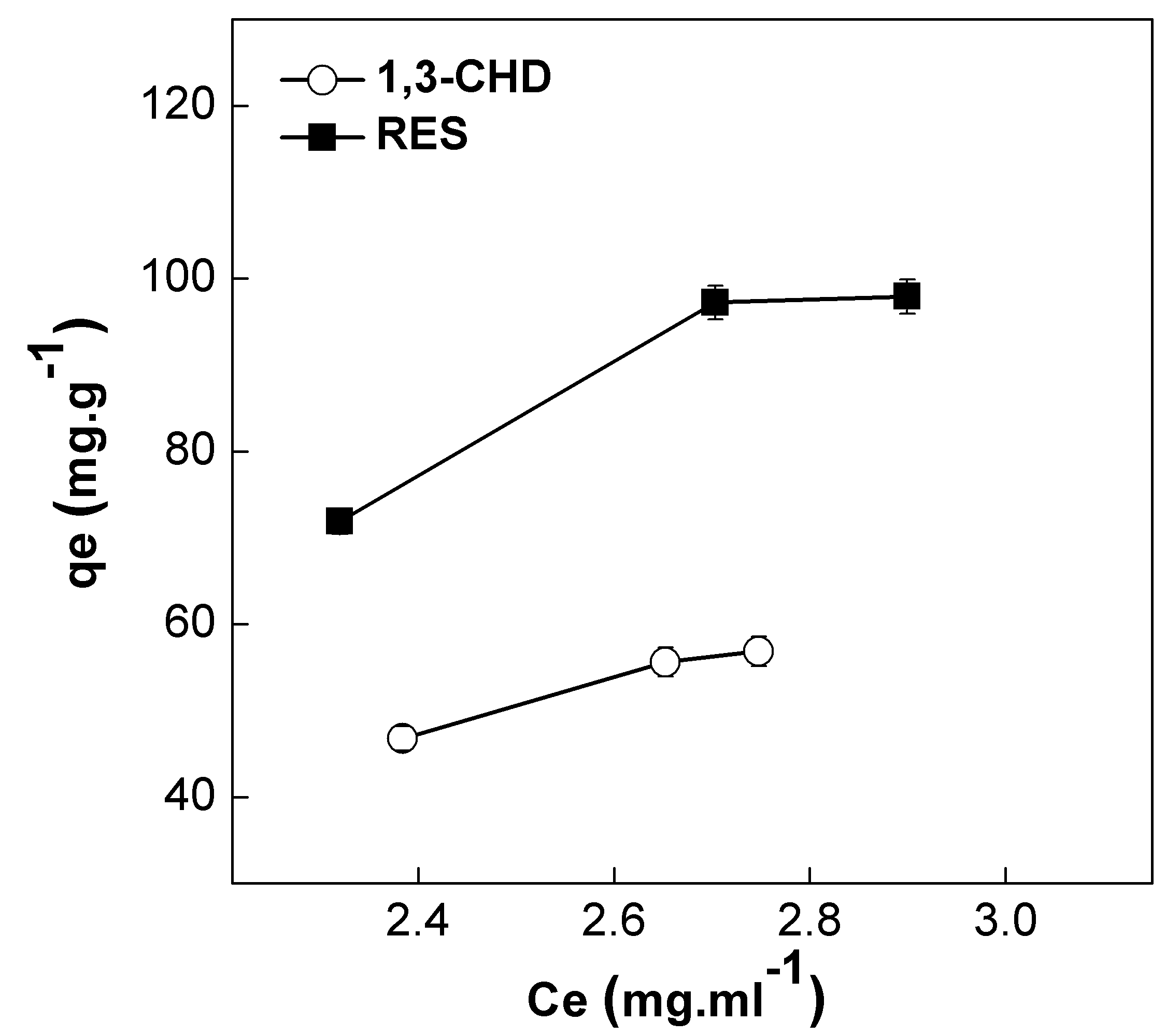


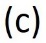

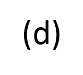

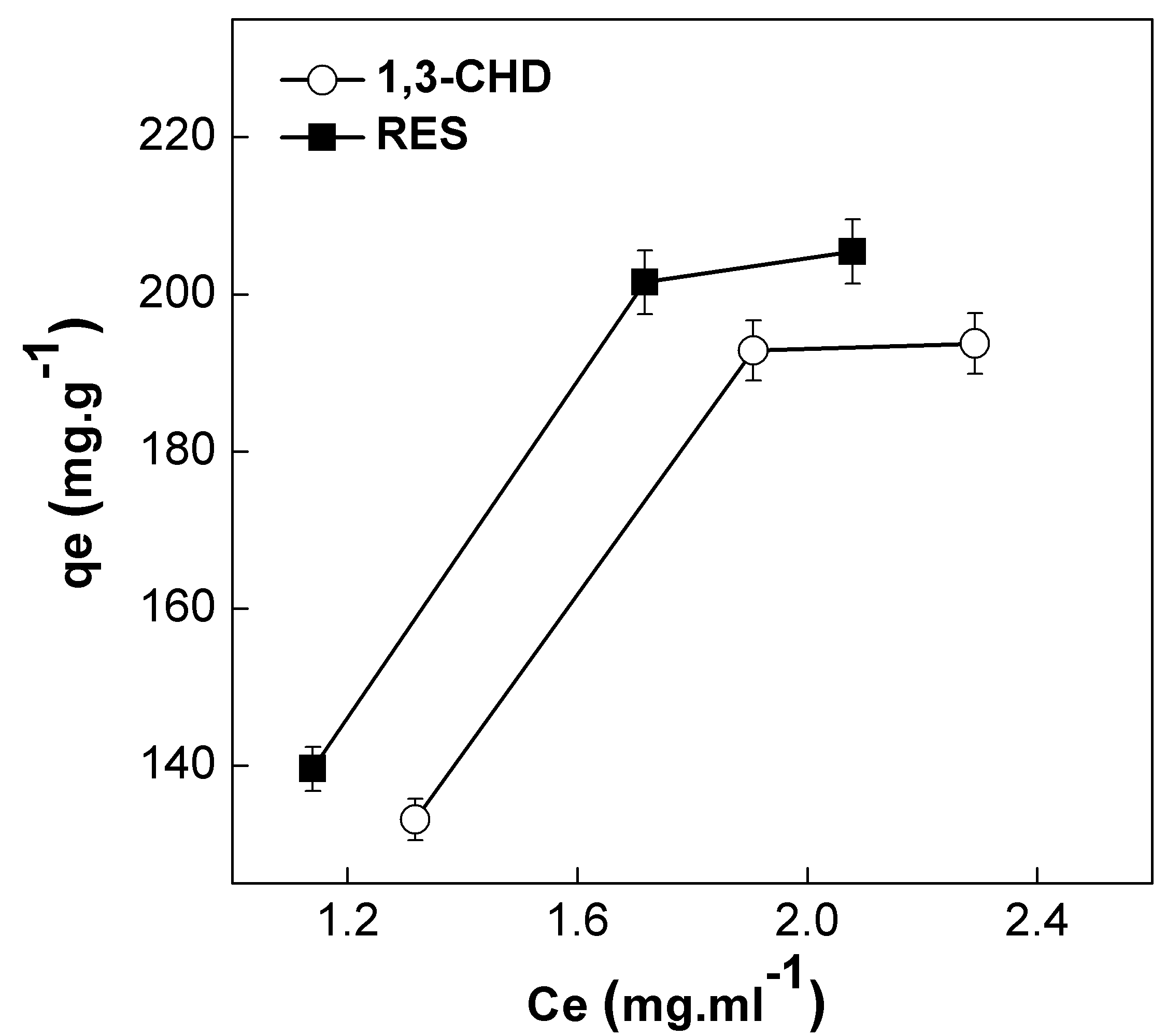

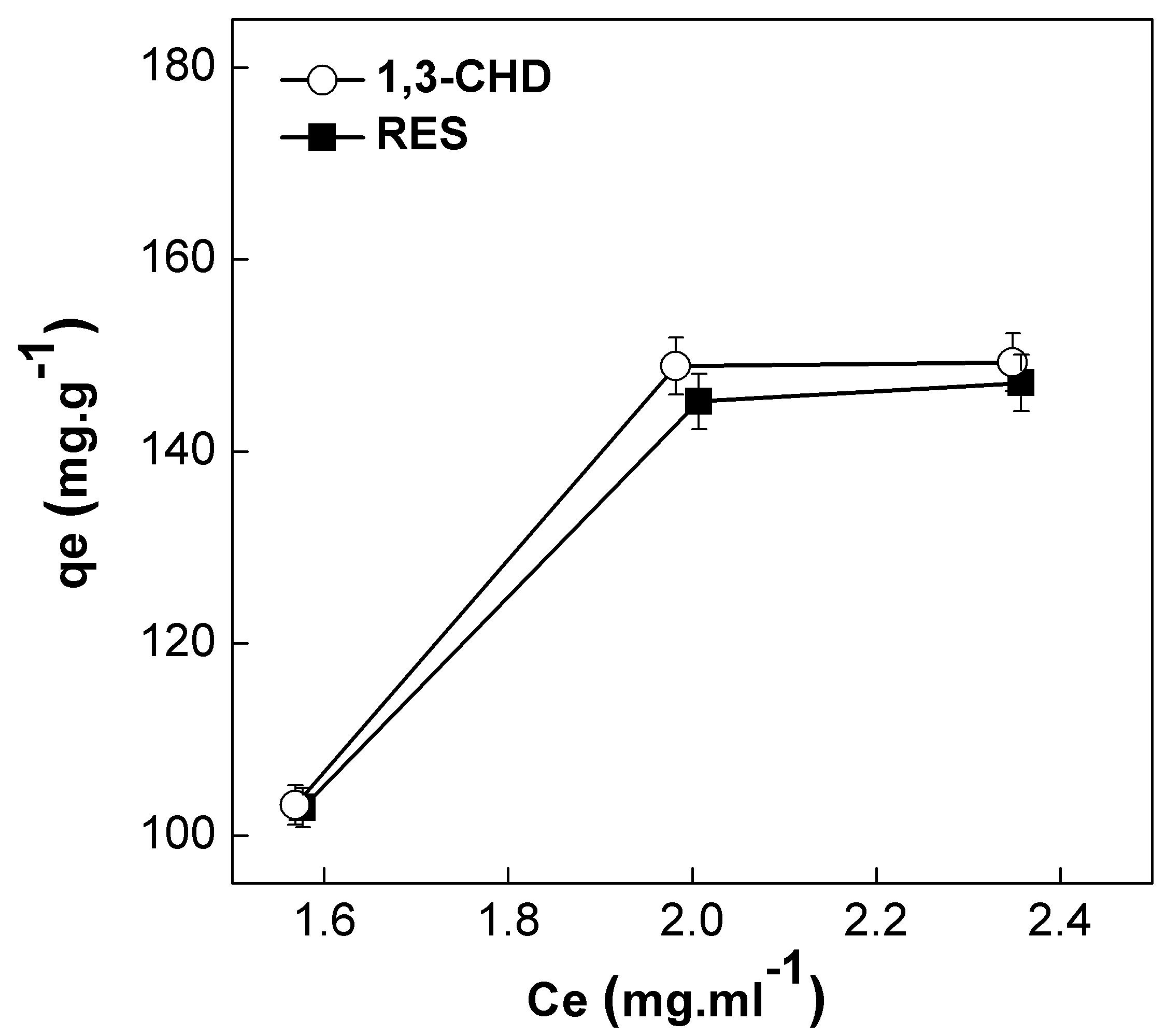


# Figure s4 Adsorption curves of resorcinol and 1,3-CHD on (a) rGO, (b) MWCNT, (c) AC, and (d) SiO_2_


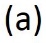

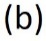

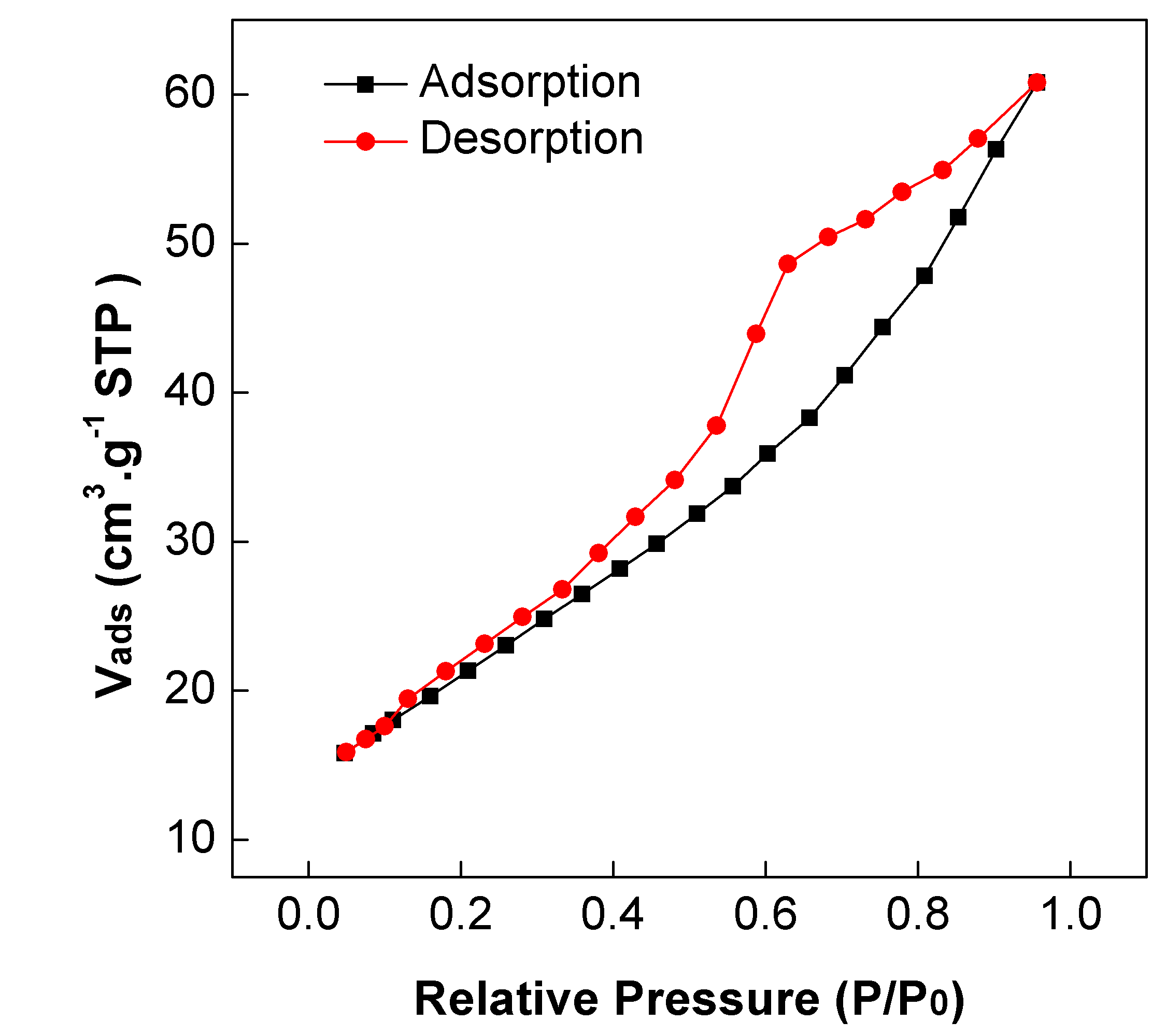

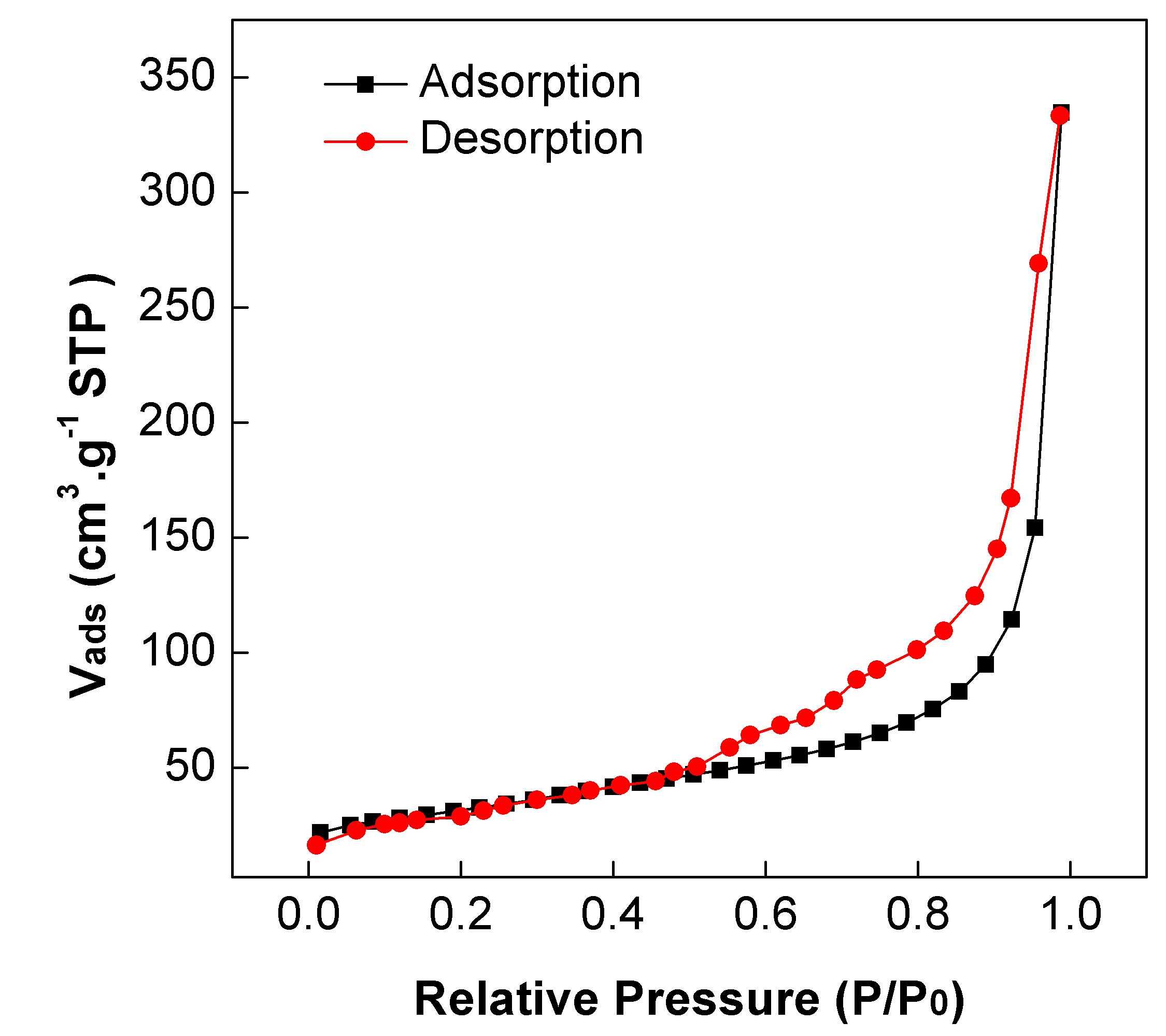


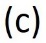

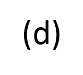

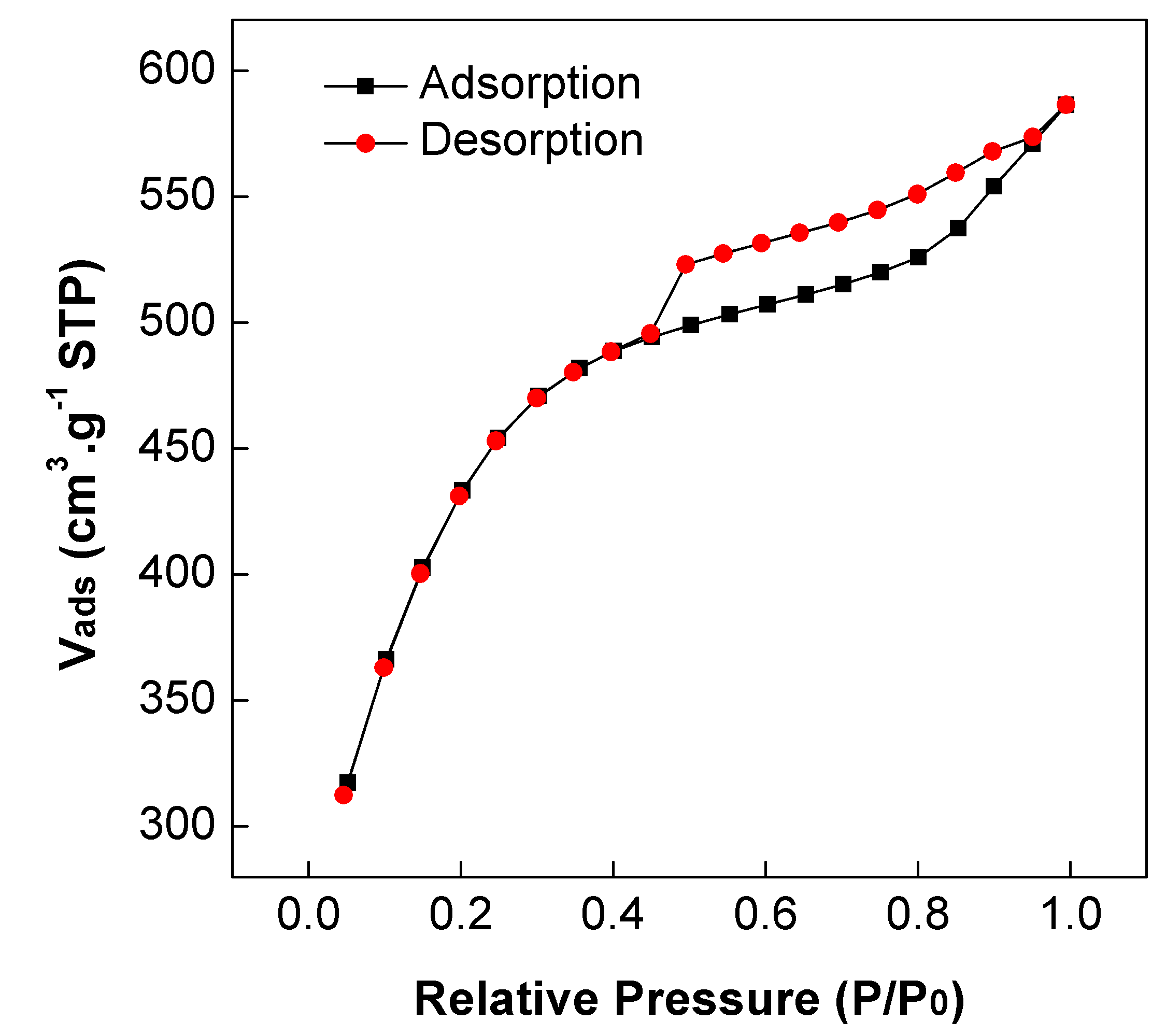

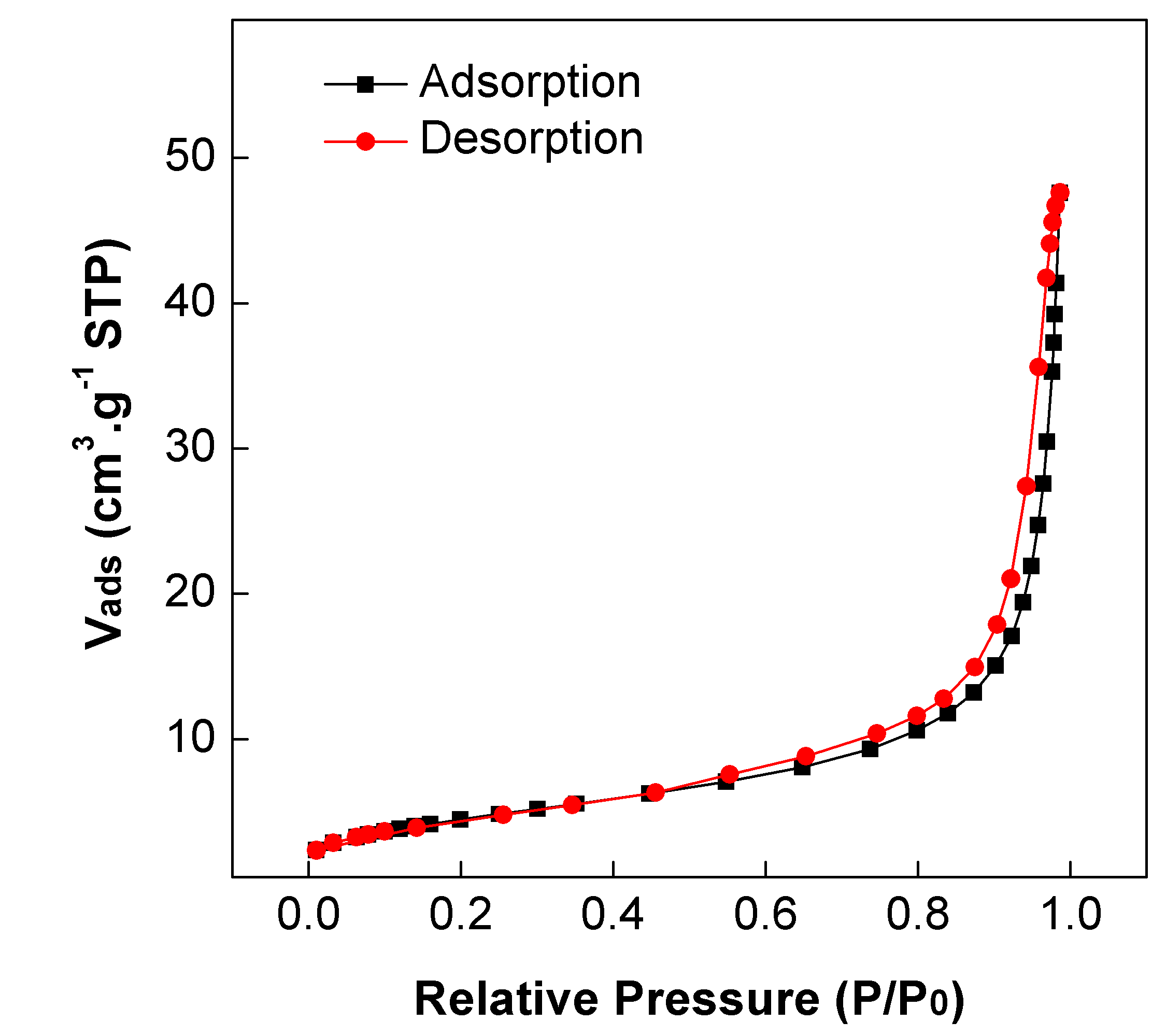


# Figure s5 Nitrogen isotherm adsorption-desorption curves of (a) Pd/rGO, (b) Pd/MWCNT, (c) Pd/AC and (d) Pd/SiO_2_


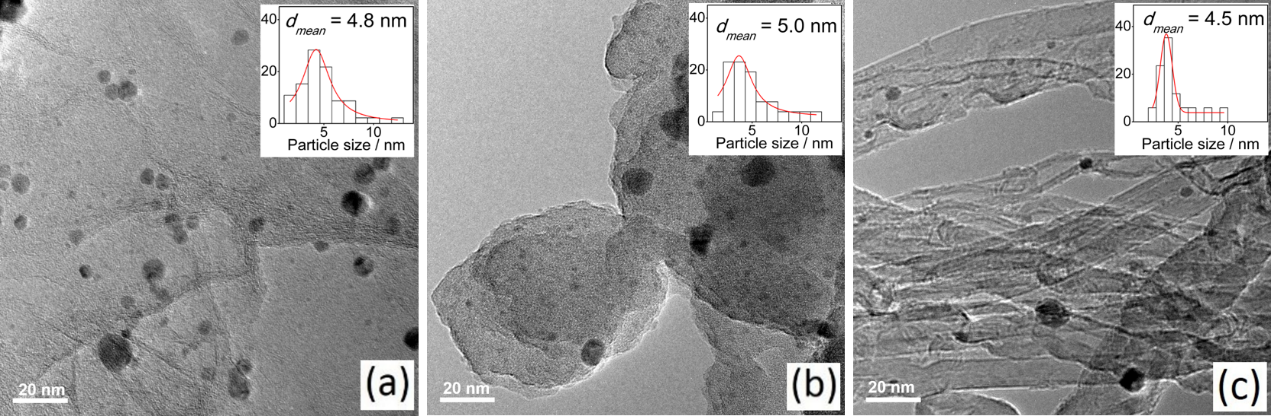


# Figure s6. TEM images of (a) Pd/AC; (b) Pd/SiO_2_ and (c) Pd/MWCNT. The average diameter of Pd particles on Pd/AC, Pd/SiO_2_, Pd/MWCNT is 4.8 nm, 5.0 nm and 4.5 nm, respectively.


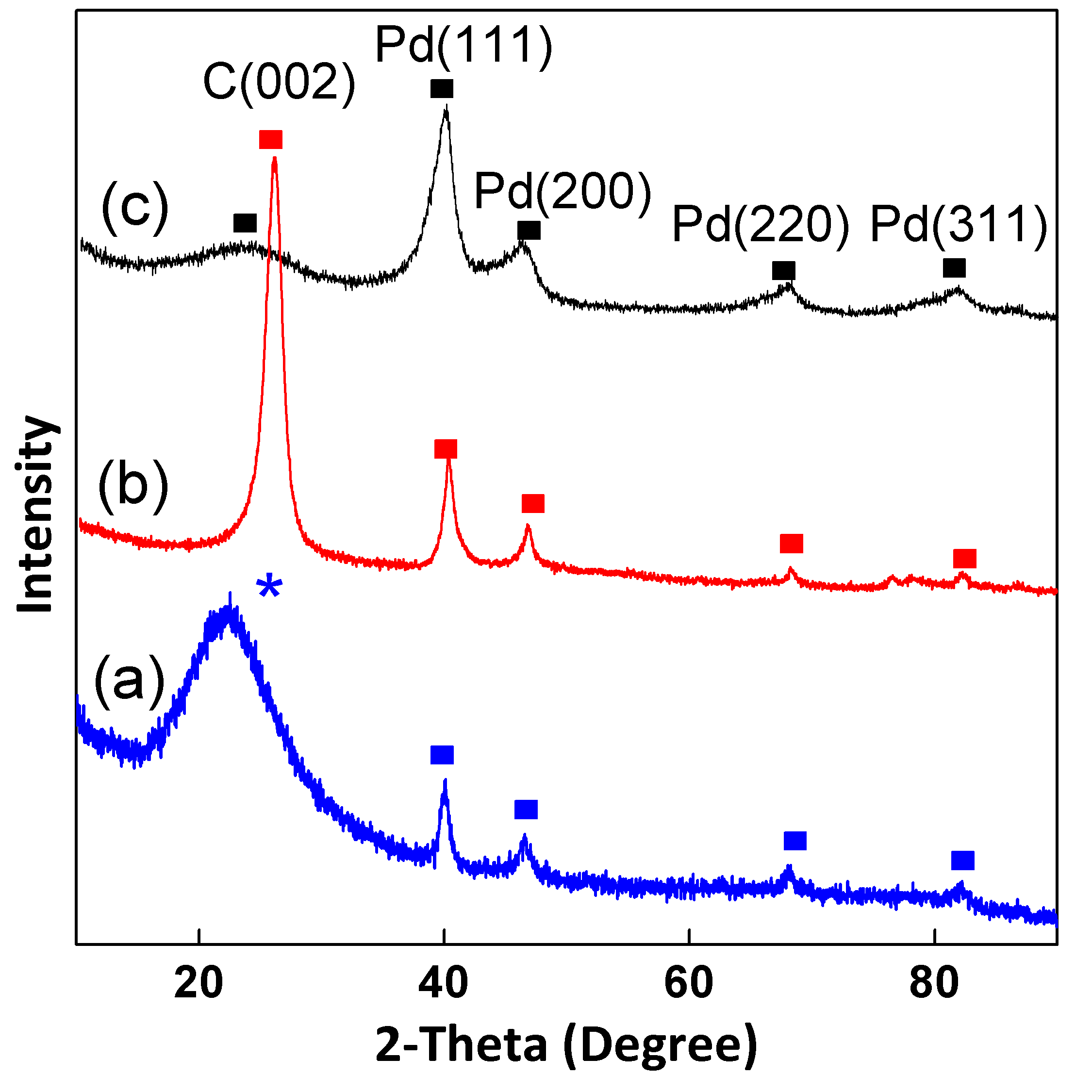


# Figure s7. XRD patterns of (a) Pd/SiO_2_; (b) Pd/MWCNT and (c) Pd/AC. The diffraction peaks at 40.1°, 46.6°, 68.1°, 82.1° correspond to Pd (111), Pd (200), Pd (220) and Pd (311), respectively. The diffraction peak at 25.1° in (c) is ascribed to the amorphous carbon of active carbon; 25.9 ° in (b) is ascribed to the 0.344 nm interlayer distance of carbon nanotube sheet in the MWCNT, and the broad peak around 2θ =21.3 ° in (a) is ascribed to the amorphous silica.
